# Measuring Policy, Systems, and Environmental Changes at Elementary Schools Involved in SNAP-Ed New Mexico Programming, 2018–2022

**DOI:** 10.5888/pcd21.230221

**Published:** 2024-01-18

**Authors:** Camille Velarde, Erica Landrau-Cribbs, Mahtab Soleimani, Theresa H. Cruz

**Affiliations:** 1 University of New Mexico Prevention Research Center, Albuquerque

## Abstract

**Introduction:**

In 2018, the New Mexico Supplemental Nutrition Assistance Program–Education (SNAP-Ed NM) incorporated policy, systems, and environmental (PSE) strategies into the state plan to increase healthy eating and physical activity. Studies of multiple PSE strategies in elementary schools are lacking.

**Methods:**

We conducted assessments of physical activity and nutrition environments at 11 elementary schools in New Mexico before and after schools were given school-specific PSE recommendations and technical assistance. Baseline data were collected in 2018 by using the School Physical Activity and Nutrition Environment Tool (SPAN-ET), which measures policy, situational, and physical environments in elementary schools. PSE scores were calculated as the proportion of criteria met within and across 27 areas of interest. Implementation of evidence-based PSE interventions began in 2019. COVID-19 school closures delayed follow-up assessments until 2022. We analyzed descriptive data to examine changes in PSE scores over time.

**Results:**

Overall mean PSE scores increased significantly from baseline (53.6%) to follow-up (62.7%). Nutrition PSE scores significantly increased by 17.6 percentage points; the policy environment showed the largest improvement (+26.0 percentage points), followed by the situational environment (+13.8 percentage points), and physical environment (+9.1 percentage points). We found a nonsignificant increase in the overall average physical activity score (+2.7 percentage points).

**Conclusion:**

Use of a standardized instrument for assessing implementation of PSE strategies across multiple schools showed significant overall improvement in nutrition scores and nonsignificant increases in physical activity scores. Providing school-specific recommendations combined with technical assistance may be an effective approach to implementing evidence-based nutrition and physical activity PSE strategies.

**Summary**

**What is already known on this topic?**

Strong evidence exists for policy, systems, and environmental (PSE) strategies that address obesity and chronic diseases by promoting healthy eating and physical activity in the school setting. PSE strategies can be implemented in low-income communities to improve equity.

**What is added by this report?**

School-specific recommendations for PSE strategies resulted in a significant increase in the number of nutrition-supportive PSEs in participating elementary schools.

**What are the implications for public health practice?**

Standardized PSE assessments, tailored evidence-based recommendations, and technical assistance can improve nutrition policies and environments at elementary schools in low-income communities.

## Introduction

Obesity among people aged 2 to 19 years in the US increased from 17.7% to 21.5% from 2011 to 2020 (1). In 2022, 28.0% of New Mexico third-grade students were obese, with substantial disparities among American Indian (46.8%) and Hispanic (28.7%) third-graders compared with their non-Hispanic White counterparts (15.9%) (2). American Indian and Hispanic children are also more likely to live in poverty (3) and have limited access to healthy foods (4) and safe places to be physically active (5) compared with non-Hispanic White children.

Childhood obesity increases the risk of chronic diseases (6), mental health concerns (7), and obesity in adulthood (8). Policy, systems, and environmental (PSE) approaches have a greater reach and impact compared with health behavior curricula alone (9), and schools play a vital role in health promotion and disease prevention efforts (10). Implementing PSE interventions in schools improves health outcomes, reduces health disparities, and enhances health equity (10).

In 2018, the New Mexico Supplemental Nutrition Assistance Program–Education (SNAP-Ed NM) incorporated PSE interventions into a state plan to increase healthy eating and physical activity. Data on the effectiveness of individual PSE interventions (eg, implementing nutrition standards for school meals, providing access to drinking water, and redesigning playgrounds to encourage physical activity during recess) are available (11,12). However, data on efforts to implement multiple PSE strategies in partnership with multiple elementary schools are not. To address this gap, the SNAP-Ed NM evaluation team developed a 3-year plan to assess PSE efforts at a sample of elementary schools. Our study aimed to determine the effectiveness of efforts to implement evidence-based PSE interventions by SNAP-Ed implementing agencies, in partnership with schools, in a real-world context. The findings have implications for future school-based PSE initiatives to increase healthy eating and physical activity in low-income communities.

## Methods

We used a quasi-experimental design wherein we selected a purposeful sample of schools to partner with SNAP-Ed implementing agencies in the testing of the implementation of PSE interventions. We conducted baseline measures in 2018 and follow-up measures in 2022 at the school level. We collected baseline measures before implementation of PSE interventions to inform potential PSE strategies for implementation and as a comparison measure for evaluation. The follow-up assessment was scheduled for 2021. The 3-year time frame allowed for implementation of changes to school policies and environments, which often takes considerable time. The COVID-19 pandemic and related school closures and restrictions delayed the follow-up assessment until 2022.

### Population

SNAP-Ed NM implementing agencies have long-standing relationships with elementary schools throughout New Mexico, where they have been providing nutrition education for more than a decade. Schools are eligible to receive SNAP-Ed programming, in general, if they meet the Community Eligibility Provision, meaning they must be located in high-poverty areas where at least 40% of students were deemed eligible for free and reduced-price meals from the National School Lunch Program in the previous year (13). Each year, SNAP-Ed NM implementing agencies provide programming for more than 150 schools.

In 2018, 5 implementing agencies contacted principals and other staff members at partner schools to discuss the opportunity to participate in a new effort to implement and evaluate PSE strategies for increasing opportunities for physical activity and healthy eating. Schools were eligible and perceived ready to participate if they received SNAP-Ed NM programming in 2018, had leadership that expressed interest, and identified at least 1 evidence-based PSE strategy that they were prepared to implement in the coming academic year. Nineteen schools met these criteria. We assessed these 19 schools at baseline and 14 at follow-up (73.7% retention rate). The 5 schools that were lost to follow-up were inaccessible because of COVID-19 pandemic–related policy changes that restricted access to school campuses. Schools participating at both time points included 11 elementary, 1 middle, and 2 high schools. We excluded the middle and high schools from the analysis because the assessment tool had not been validated in these settings. The final sample comprised 11 elementary schools.

### Intervention

We conducted the baseline PSE assessment and provided the results to SNAP-Ed implementing agencies and school leadership with recommendations for PSE changes specific to their school context. No single evidence-based PSE strategy was implemented across all schools. Recommendations varied by school and included such evidence-based strategies as strengthening written wellness policies, developing school gardens, teaching nutrition education in all grades, having an active wellness committee, and increasing the amount of portable equipment (eg, balls, jump ropes, hula hoops) for active play. Schools chose and implemented selected strategies at their own pace with technical assistance from SNAP-Ed implementing agencies. Schools obtained funding for strategies with real costs (eg, school gardens, shade structures), often with the assistance of SNAP-Ed implementing agencies. Funding came from various sources, including the federal government, foundations, and community donations. The unanticipated COVID-19 pandemic–related school closures and restrictions in the 2019–2020 and 2020–2021 academic years disrupted implementation and affected both existing and new PSE strategy selection and implementation.

### Instrument

We used a validated instrument, the School Physical Activity and Nutrition Environment Tool (SPAN-ET) (14), to conduct an assessment of PSE interventions at participating schools. We measured effectiveness as the extent to which SPAN-ET PSE scores increased over time in schools. SPAN-ET is a reliable instrument for evaluating the physical activity and nutrition environments in elementary schools (15). It provides comprehensive data to assess the school environment consistently and identify areas affecting obesity-preventing behaviors among students. The SPAN-ET comprises 27 areas of interest (AOIs) that evaluate the physical, situational, and policy environments related to physical activity and nutrition (Figure). Each AOI is assessed via multiple criteria, and each criterion is assigned a value of 0 (not met) or 1 (met). These scores are then summed and divided by the total number of criteria to attain scores represented as the proportion of criteria met within each AOI, domain, and category; the scores are presented as percentages ranging from 0% to 100%. We added criteria to 2 SPAN-ET AOIs: 1) assessing sustainability resources for existing garden spaces or greenhouses (AOI 18: nutrition–garden features) and 2) documenting social marketing materials and nutrition education areas and equipment (AOI 20: nutrition–food and beverage habits). We added these items to provide a more comprehensive assessment based on the specific PSE strategies and goals of the implementing agencies working in collaboration with the schools. Although the instrument developer made some modifications to the original SPAN-ET between 2018 and 2022, we assessed only items that were included in both years.

**Figure:**
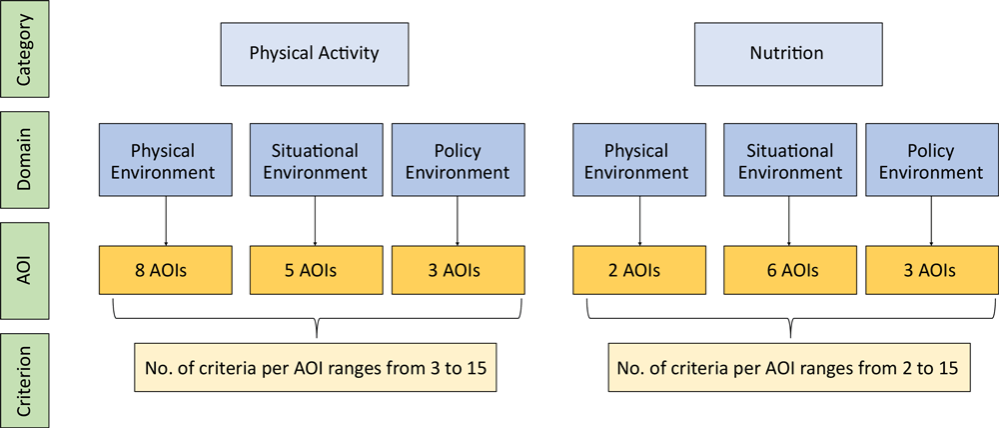
The categories, domains, areas of interest (AOIs), and number of criteria per AOI in the School Physical Activity and Nutrition Environment Tool (SPAN-ET).

SPAN-ET has 3 key domains: physical environment, situational environment, and policy environment.

**Physical environment.** The physical environment domain includes built environment features in and around the school that contribute to physical activity or nutrition. Eight AOIs assessed the physical environment for physical activity, including gymnasiums, outdoor play areas, shade structures, natural features, school gardens, and neighborhood features. Two AOIs assessed the physical environment for nutrition: the cafeteria and school gardens.

**Situational environment.** The situational environment domain includes the use of the physical environment, such as the appearance and atmosphere of the space and the promotion of actions or activities in the space. Five AOIs assessed the situational environment for physical activity: portable equipment, spaces that stimulate the senses, features that promote physical activity, opportunities to be active in before- and after-school programs, and landscape/garden spaces that promote physical activity. Six AOIs assessed the situational environment for nutrition, including school meals, food habits and practices, availability of water, cafeteria atmosphere, and extracurricular activities.

**Policy environment.** The policy environment domain includes wellness policies, wellness committees and objectives, and policies on hiring trained staff. Three AOIs assessed the policy environment for physical activity, including physical activity wellness policy, a wellness committee, and structured physical education policies. Three AOIs assessed the policy environment for nutrition, including nutrition wellness policy, wellness committee, and health and nutrition education.

### Data collection

Per SPAN-ET protocol, the assessments included a document review (eg, school wellness policies, meal menus, parent handbooks), on-site observations of facilities (eg, cafeteria, gymnasium, playground, physical education class), and interviews with school administrators and staff members about AOIs, including school staffing, curricula, and policies. Before each data collection period (2018 and 2022), the data collection team, which included both the research team and SNAP-Ed implementing agencies, were rigorously trained on the instrument and methods, beginning with a 6-hour class conducted by the developer of the tool. After the classroom training, the research team participated in internal reviews and trainings and conducted a training assessment at a school that was not part of the study to gain experience with the tool.

The research team then systematically analyzed policies and other relevant written materials by using standardized scoring criteria in the SPAN-ET. In both 2018 and 2022, document review was followed by a day-long visit to each school for observations and structured interviews, with criteria scored per SPAN-ET protocols. All assessments were scored independently by 2 data collectors, who met to reconcile scores until consensus was achieved. Any scoring differences were resolved by examining the original data (eg, observational photographs and notes, policy documents, interview transcripts). If further clarification was needed to reach 100% agreement, data collectors revisited the site or recontacted school personnel. Incorporating rigorous training, opportunities to practice on-site at schools, quality improvement checks, observational photographs, and meetings to reconcile assessments supported strong interrater reliability.

### Data analysis

For each school in 2018 and 2022, we collected data on the number of students enrolled, the percentage of students eligible for free or reduced-price lunch through the National School Lunch Program, the percentage of Hispanic students, and the percentage of American Indian students. We obtained these data from the New Mexico Public Education Department (16) and the National Center for Education Statistics (17).

We calculated the proportion of met criteria for each AOI and averaged scores across schools within each domain. We calculated the overall proportion of met criteria for the physical activity and nutrition domains. Mean scores were calculated for each AOI across schools and transformed into a percentage representing the number of schools that met criteria. Within schools, the proportion of met AOIs in physical activity and nutrition categories was calculated, resulting in an overall score for each school with a maximum of 100%. A paired-samples *t* test assessed differences between mean baseline and follow-up scores for each domain and category.

## Results

At baseline, 10 of 11 student populations at participating schools were majority Hispanic and all students at 8 of 11 schools were eligible for free or reduced-price lunch through the National School Lunch Program (Table 1). Student enrollment ranged from 75 to 616 at baseline.

**Table 1:** Demographic Characteristics at Baseline and Follow-Up of a Sample of 11 Elementary Schools in New Mexico Participating in the SNAP-Ed PSE Evaluation, 2018 and 2022^a^

School	Student enrollment, no.		Students eligible for free or reduced-price lunch, %		Hispanic students, %		American Indian students, %	
	Baseline (2018)	Follow-up (2022)	Baseline (2018)	Follow-up (2022)	Baseline (2018)	Follow-up (2022)	Baseline (2018)	Follow-up (2022)
ES1	99	98	100	100	93	98	1	≤5
ES2	298	302	69	100	78	80	2	2
ES4	559	521	67	100	72	71	2	2
ES5	420	384	88	100	85	86	0	≤1
ES7	79	80	100	100	0	0	100	100
ES8	75	78	100	100	64	68	0	≤5
ES9	552	541	100	100	62	62	1	≤1
ES10	296	279	100	100	67	69	0	≤2
ES11	616	616	100	100	76	75	0	≤1
ES12	394	377	100	100	73	72	3	≤5
ES16	307	303	100	100	69	70	1	≤1

Abbreviations: ES, elementary school; PSE, policy, systems, and environment; SNAP–Ed, Supplemental Nutrition Assistance Program–Education.

a Sources: New Mexico Public Education Department (16); National Center for Education Statistics (17).

### Change in proportion of criteria met among all schools

We found no significant differences in mean overall physical activity scores from baseline to follow-up for the 3 physical activity domains across all schools (Table 2). Two nutrition scores increased significantly from baseline to follow-up: nutrition–situational environment, which increased from 60.9% to 74.7% (+13.8 percentage points; *P* = .005), and nutrition–policy environment, which increased from 28.9% to 54.9% (+26.0 percentage points; *P* <.001). The overall score for nutrition PSEs increased significantly by 17.6 percentage points, from 50.8% to 68.5% (*P* < .001).

**Table 2:** Change in Mean Scores^a^ From Baseline to Follow-Up, by SPAN-ET Domain and Category, in a Sample of 11 Elementary Schools in New Mexico, 2018 and 2022

Domain	Category							
	Physical activity				Nutrition			
	Baseline score (2018)	Follow-up score (2022)	Percentage-point change in score	*P* value^b^	Baseline score (2018)	Follow-up score (2022)	Percentage-point change in score	*P* value^b^
Physical environment	64.4	70.2	+5.8	.21	72.7	81.8	+9.1	.07
Situational environment	53.7	56.5	+2.8	.67	60.9	74.7	+13.8	.005
Policy environment	40.2	36.0	−4.2	.50	28.9	54.9	+26.0	<.001
Overall^c^	55.7	58.3	+2.7	.44	50.8	68.5	+17.6	<.001

Abbreviation: SPAN-ET, School Physical Activity and Nutrition Environment Tool.

a Reported as the percentage of schools that met criteria.

b Paired samples *t* test conducted to compare mean scores from baseline to follow-up; significance set at *P* ≤ .05.

c Mean score across all 11 schools for the category, including all 3 domains.

The highest average increase in mean scores (+30.3 percentage points) from baseline to follow-up among AOIs across all schools was in the physical activity–physical environment domain, AOI 3, which addresses shelter and shade structures (Table 3). The highest average decrease (−5.5 percentage points) in this domain was for AOI 1, which addresses gymnasiums and dedicated multipurpose spaces for physical activity. The highest average increase (+12.1 percentage points) in the physical activity–situational environment domain was for AOI 13, gardening activity spaces and programs. The highest average decrease (−12.7 percentage points) in this domain was for AOI 9, portable equipment. The highest average increase (+56.4 percentage points) in the physical activity–policy environment domain was for AOI 15, which addresses physical activity and wellness committees. The highest average decrease (−44.4 percentage points) in this domain was for AOI 16, structured physical education. The highest average increase (+13.6 percentage points) in the nutrition–physical environment domain was for AOI 18, school garden features. We found no decreases from baseline to follow-up in this domain. The highest average increase (+22.1 percentage points) in the nutrition–situational environment domain was for AOI 20, promoting healthy food and beverage habits. We found no decreases in this domain. The highest average increase (+78.2 percentage points) in the nutrition–policy environment domain was for AOI 26, which addresses nutrition and wellness committees. The highest average decrease (−6.8 percentage points) in this domain was for AOI 27, nutrition education.

**Table 3:** Change in Mean Scores^a^ From Baseline to Follow-Up, by SPAN-ET Area of Interest (AOI), in a Sample of 11 Elementary Schools in New Mexico, 2018 and 2022

AOI no./description	Mean score		
	Baseline (2018)	Follow-up (2022)	Percentage-point change
**Category: physical activity**			
**Domain: physical activity–physical environment**			
1. Gymnasium and/or dedicated multipurpose space is available to accommodate physical education, physical activity/active play.	68.5	63.0	−5.5
2. Outdoor space is adequately sized for teaching and physical activity, has clearly defined boundaries, and comprises a variety of appropriate activity settings, fixed equipment, and materials.	69.7	77.8	+8.1
3. Shade (natural and/or artificial structures) and/or shelters provide protection from sun and/or inclement weather.	33.3	63.6	+30.3
4. Natural or green playground areas, elements, and/or features are available.	38.6	56.8	+18.2
5. Gardens and landscaping includes a variety of plantings, growing environments (eg, orchards, inground beds, raised beds, and/or containers), and topical conditions.	27.3	45.5	+18.2
6. Indoor and outdoor surfaces and surface markings support movement and activity variety and safety.	84.1	79.5	−4.5
7. School yard, grounds, and outdoor facilities are enclosed and safe for physical activity.	90.9	96.1	+5.2
8. Built environment features and neighborhood proximal to the school property provide safe physical activity/active transportation access for pedestrian and bicycle circulation from the neighborhood to the site entrances to the building.	50.9	63.6	+12.7
**Domain: physical activity–situational environment**			
9. Portable equipment is available, easily accessible, and offers a wide variety/range of experiences.	58.2	45.5	−12.7
10. Indoor and outdoor spaces have a friendly, welcoming, inclusive, and inviting atmosphere that is culturally appropriate and stimulates the senses (ie, touch/textures, smell, listening, looking, vestibular and proprioceptive input).	67.5	76.6	+9.1
11. Indoor and outdoor fixed and portable features promote physical activity, active play, and a variety of developmental movements.	68.2	74.2	+6.1
12. School supports and/or partners with community resources to provide physical activity opportunities before and/or after school and in the summer. Extracurricular programs are available in various indoor and outdoor facilities.	43.0	44.6	+1.7
13. Existing landscape/garden spaces are designated and used to promote physical activity/active lifestyle habits.	24.2	36.4	+12.1
**Domain: physical activity–policy environment**			
14. School has implemented the district wellness policy, drafted a written physical activity policy, and communicated with school staff, families, and the district regarding students’ physical activity progress on an annual basis; school’s physical activity goals are integrated into the school’s overall long-range wellness goals/plan.	25.5	27.3	+1.8
15. Active wellness council/committee exists that has specific physical activity–related objectives and/or an active physical activity council/subcommittee.	3.6	60.0	+56.4
16. School has a structured physical education/physical activity program that is coordinated and/or instructed by trained/credentialed physical educator(s).	76.8	32.3	−44.4
**Category: nutrition**			
**Domain: nutrition–physical environment**			
17. Cafeteria or alternative meal service area (ie, classroom) offers a clean, pleasant, and safe setting with adequate space for eating meals.	90.9	98.2	+7.3
18. School has orchards, greenhouses, in-ground gardens, raised beds, and/or container gardens to grow edible produce.	27.3	40.9	+13.6
**Domain: nutrition–situational environment**			
19. Program meets or exceeds food and nutrition standards and is managed efficiently and inclusively.	71.7	79.8	+8.1
20. Promoting healthy food and beverage choices and habits is accepted and integrated into the school culture.	50.6	72.7	+22.1
21. All foods and beverages served or sold outside of the school meals program during the regular and extended school day meet or exceed federal and/or state standards for foods and beverages sold in schools.	52.7	74.5	+21.8
22. Clean, safe, palatable drinking water is available, accessible, and promoted to all students and staff throughout the school day.	75.0	75.0	0
23. Meals served to students are attractively presented in a pleasant (friendly, comfortable, and inviting) environment with sufficient time for eating.	71.8	88.2	+16.4
24. School provides and/or partners with community resources to provide healthy foods and beverages, and nutrition education opportunities before and/or after school and in the summer.	71.8	88.2	+16.4
**Domain: nutrition–policy environment**			
25. School has implemented the district wellness policy, drafted a written nutrition policy, and communicates with school staff, families, and the school district regarding its nutrition progress on an annual basis. The school’s nutrition goals are integrated into the school’s overall long-range wellness improvement goals/plan.	27.3	53.3	+26.1
26. Active wellness council/committee exists and has specific nutrition-related objectives and/or an active nutrition council/subcommittee.	3.6	81.8	+78.2
27. Health education program includes functional knowledge and skills-based nutrition lessons. Nutrition behaviors/habits are taught in all grades.	47.7	40.9	−6.8

Abbreviation: SPAN-ET, School Physical Activity and Nutrition Environment Tool.

a Reported as the percentage of schools that met criteria.

### Change in mean school scores from baseline to follow-up

We observed a significant increase (+9.1 percentage points) in overall scores from baseline to follow-up: from 53.6% at baseline to 62.7% at follow-up (*P* = .02) (Table 4). We found a significant increase (+17.6 percentage points) in average nutrition scores from baseline to follow-up, from 50.8% at baseline to 68.5% at follow-up (*P* < *.*001). We found no significant differences in physical activity scores from baseline to follow-up (*P* = .44).

**Table 4:** Change in Overall Mean SPAN-ET Scores From Baseline to Follow-Up in a Sample of 11 Elementary Schools, 2018 and 2022

School	Overall score^a^			Overall physical activity			Overall nutrition		
	Baseline (2018)	Follow-up (2022)	Percentage-point change	Baseline (2018)	Follow-up (2022)	Percentage-point change	Baseline (2018)	Follow-up (2022)	Percentage-point change
ES1	49.7	61.0	+11.2	49.1	55.7	+6.6	50.6	67.9	+17.3
ES2	61.5	64.7	+3.2	65.1	66.0	−0.9	56.8	63.0	+6.2
ES4	66.8	52.9	−13.9	76.4	55.7	−20.1	54.3	49.4	−4.9
ES5	48.1	59.4	+11.2	52.8	49.1	−3.8	42.0	72.8	+30.9
ES7	56.1	55.1	−1.1	53.8	52.8	+14.2	59.3	69.1	+9.9
ES8	39.6	57.8	+18.2	38.7	44.3	−9.4	40.7	64.2	+23.5
ES9	55.1	77.5	+22.5	58.5	73.6	+15.1	50.6	82.7	+32.1
ES10	57.2	69.0	+11.8	60.4	64.2	+3.8	53.1	75.3	+22.2
ES11	51.3	70.1	+18.7	50.0	64.2	+14.2	53.1	77.8	+24.7
ES12	53.5	66.8	+13.4	53.8	58.5	+4.7	53.1	77.8	+24.7
ES16	50.3	55.6	+5.3	53.8	57.6	+3.8	45.7	53.1	+7.4
All	53.6	62.7	+9.1^b^	55.7	58.3	+2.7	50.8	68.5	+17.6^c^

Abbreviations: ES, elementary school; SPAN-ET: School Physical Activity and Nutrition Environment Tool.

a Overall score combines the physical activity and nutrition categories for each school.

b *P* = .02; paired-samples *t* test conducted to compare overall mean scores from baseline to follow-up for all 11 schools; significance set at *P* ≤ .05.

c *P* < .001; paired samples *t* test conducted to compare overall nutrition mean scores from baseline to follow-up for all 11 schools; significance set at *P* ≤ .05.

## Discussion

Our study aimed to determine the effectiveness of efforts to improve nutrition and physical activity policies, systems, and environments across multiple elementary schools in a real-world context. Our results demonstrated a significant increase in elementary school nutrition policies and environments during the 4-year intervention period using a standardized PSE assessment. This study was novel in its examination of changes in SPAN-ET PSE scores across 11 schools implementing evidence-based strategies of their choosing following school-specific recommendations combined with technical assistance from SNAP-Ed implementing agencies. Our findings are consistent with other research showing that identifying gaps, making recommendations, and providing technical assistance can improve adoption of new policies or practices in the school environment (18).

Among the 11 participating elementary schools in our study, we observed a significant increase of 17.6 percentage points in the overall nutrition score across schools. This increase was largely driven by the addition of active wellness councils and nutrition policies, improved access to healthier foods outside school meals, and an increase in the number of school gardens. Wellness councils and nutrition policies can improve the healthfulness of foods consumed by students in the school environment (19). Additionally, school gardens can improve student health behaviors and outcomes, particularly among students facing economic disadvantages (20), which can lead to increases in health equity. In addition to recommendations for and technical assistance with these PSE strategies, decisions by participating schools to implement new nutrition policies and improved nutrition environments may have been influenced by the increased recognition of food insecurity among students during the COVID-19 pandemic (21).

In our research, none of the physical activity domains showed significant increases in PSE scores. However, scores increased substantially among some AOIs. For example, we noted several improvements to the outdoor environment, including improvements to playground areas, development of school gardens, and the addition of shade structures. Research indicates that gardening, coupled with food preparation, nutrition education, and physical activity instruction, can improve children’s vegetable intake and physical activity levels (22). Additionally, providing shade in outdoor areas can substantially promote use of play areas, reduce sun exposure, and prevent heat-related injury (23). The COVID-19 pandemic produced an unexpected opportunity as funding to construct shade structures was made available to support outdoor learning environments. These structures continue to provide a safe, comfortable area for outdoor active play among school students.

We observed major declines in both the nutrition and physical activity categories in the teaching of nutrition and physical education. Research suggests that education on healthy lifestyles results in improvements in nutrition and physical activity among students (24). However, consistent with the national literature (25), participating schools reported difficulty in maintaining qualified staff, including health and nutrition educators and trained physical education teachers (26), during the COVID-19 pandemic. Additionally, schools restricted access to nonschool personnel during the pandemic, limiting access by SNAP-Ed nutrition educators. Declines in PSE scores in these areas may rebound as schools work to recruit qualified physical education and health staff and return to permitting access to external nutrition education programming.

Additionally, our results showed that PSE supports for physical activity declined unexpectedly from baseline to follow-up in 2 areas. These were access to a gymnasium for physical activity and active play and the availability of portable play equipment. Research demonstrates that students, particularly girls, with access to portable equipment engage in more moderate to vigorous physical activity than students without such access (27). Declines in scores in these areas may be attributable to the COVID-19 pandemic, which resulted in some schools closing gymnasiums in favor of outdoor play and some schools limiting use of portable play equipment due to the additional cleaning requirements.

Our research also demonstrated the successful implementation of evidence-based PSE strategies in schools characterized by disproportionately high populations of Hispanic students and American Indian students, where nearly all students qualified for the National School Lunch Program. In the US, people with low socioeconomic status, as well as those identifying as Hispanic or American Indian, have an elevated prevalence of obesity and other chronic health conditions (28,29). SNAP-Ed NM purposefully focuses programming, including PSE intervention efforts, on schools located in low-income communities that, in New Mexico, predominantly serve Hispanic and American Indian students. Effective PSE efforts in these schools can improve physical activity and nutrition, reduce health disparities linked to obesity, and improve health equity (12).

Our study was designed in 2018, and interventions began in 2019. The disruptions caused by the COVID-19 pandemic were unanticipated and had a substantial effect on the study. These disruptions altered the timing and implementation of PSE strategies, ultimately affecting scores and effectiveness. Some scores were reportedly positively influenced (eg, shade structures) while others were negatively influenced (eg, recruitment and retention of qualified physical education teachers). The pandemic also introduced additional factors, including the mental health of staff members and students, workforce changes, and limited access to resources, which may have influenced engagement in PSE implementation. Despite the pandemic, we observed improvements in most nutrition and physical activity PSEs over time.

### Strengths and limitations

Study strengths include the use of a standardized instrument to assess school-level nutrition and physical activity PSEs and identify gaps for strategic intervention. Additionally, using the SPAN-ET to evaluate SNAP-Ed programming across multiple schools provided a standardized way to measure and assess PSE implementation across the state. This tool and standardized protocol allowed us to capture changes over time, offering insights into the effectiveness of efforts to implement evidence-based strategies. We also incorporated a rigorous training protocol and reconciliation process to enhance reliability. The study also capitalized on existing partnerships between nutrition educators and schools and provided flexibility for schools to identify the PSE strategies that they were interested in pursuing in a real-world context. We provided school leadership with a detailed breakdown of scores and school-specific recommendations for PSE strategies to inform their choices.

Our study has several limitations. It lacked a standard intervention across sites; each school chose the evidence-based PSE strategies that they determined were important and feasible to implement. However, our approach aligns with principles of community-engaged research and implementation science. Additionally, the absence of a comparison group or control condition makes it challenging to establish causality or differentiate the effects of implementation efforts from other factors, including the COVID-19 pandemic. Some of these limitations could be addressed by examining implementation in the postpandemic period, incorporating an appropriate control group, and measuring the dissemination and implementation of specific strategies over time. Additionally, the COVID-19 pandemic and resultant school district policies precluded capturing data from all 19 original sites. These additional data may have altered the findings if the schools that did not participate in the follow-up assessment had differed in their implementation of PSE strategies.

### Implications

Standardized PSE assessments, combined with tailored evidence-based recommendations and technical assistance, are effective at improving nutrition policies and environments in elementary schools in low-income communities. Additionally, the SPAN-ET can be used to evaluate changes over time in the nutrition and physical activity PSEs in place across multiple schools. Because it is a standardized, reliable tool, comparisons can also be made across studies.

### Conclusion

Use of the SPAN-ET, both for identifying potential PSE strategies and evaluating SNAP-Ed programming across multiple schools in the state, revealed significant overall improvement in nutrition scores and nonsignificant increases in physical activity scores. We found considerable variation in the pre–post changes in score among AOIs: these changes ranged from a decrease of 44.4 percentage points for having a physical education program coordinated by a trained physical educator to an increase of 78.2 percentage points for having an active wellness committee with nutrition objectives. Providing school-specific recommendations combined with technical assistance may be an effective approach to implementing evidence-based nutrition and physical activity PSE strategies in real-world contexts. However, the COVID-19 pandemic affected the types of strategies implemented, the timing of implementation, and the timing of data collection for evaluation. Future research may include use of a comparison group to assess the causal effect of the interventions. However, some evidence suggested that the collection of baseline data itself influenced the intentions of school staff to implement PSE changes. Dissemination and implementation studies would also be useful to answer research questions about the characteristics of schools or school leadership that adopt different strategies, the fidelity with which the interventions are implemented, and whether interventions are sustained over time and with new school leadership.
